# In Vitro Effects of Amygdalin on Proliferation and Apoptosis in SH-SY5Y Neuroblastoma Cells

**DOI:** 10.3390/cimb48050522

**Published:** 2026-05-17

**Authors:** Tuba Gül, Mücahit Seçme

**Affiliations:** 1 Department of Neurology, School of Medicine, Ordu University, 52200 Ordu, Türkiye; tubayazici@odu.edu.tr; 2 Department of Medical Biology, School of Medicine, Ordu University, 52200 Ordu, Türkiye

**Keywords:** amygdalin, neuroblastoma, SH-SY5Y cells, apoptosis, cell invasion

## Abstract

Background and Objectives: Neuroblastoma represents the most common extracranial solid tumor in childhood and is associated with a poor prognosis in high-risk cases. Amygdalin, a naturally occurring cyanogenic glycoside, has been reported to exhibit anti-tumor properties in various cancer models; however, its effects on neuroblastoma cells remain insufficiently characterized. The present study was conducted with the objective of investigating the effects of amygdalin on cell proliferation, apoptosis, and invasion in SH-SY5Y neuroblastoma cells in vitro. Materials and Methods: The SH-SY5Y neuroblastoma cells were cultivated under the optimal conditions for their growth. The cytotoxic effect of amygdalin was determined using the CCK8 assay, which is dose- and time-dependent. Total RNA isolation was performed using Trizol. Subsequently, a process of cDNA synthesis was initiated. The real-time PCR method was utilized to ascertain alterations in the expression levels of mRNA molecules associated with apoptosis, namely Bax, Bcl2, caspase-3, caspase-7, caspase-8, caspase-9, caspase-10, NFkB, and invasion-related genes MMP2, MMP9, TIMP1, and TIMP3. Furthermore, alterations in NFkB levels were examined through the utilization of the ELISA method. Results: The IC50 value of amygdalin in SH-SY5Y cells was determined to be 112.7 µM at 24 h. Amygdalin demonstrated a dose-dependent cytotoxic effect on neuroblastoma cells. Furthermore, the study revealed that the drug induced apoptosis through the upregulation of BAX and BID, and the downregulation of BCL-2 and NF-κB. This process led to a reduction in cell proliferation. Furthermore, the study demonstrated an anti-invasive effect through the downregulation of MMP9 and the upregulation of TIMP1 and TIMP3. In addition, a substantial decrease in NF-κB protein concentration was observed. Conclusions: These findings demonstrate that amygdalin exerts anti-proliferative, pro-apoptotic, and anti-invasive effects in SH-SY5Y neuroblastoma cells in vitro. Amygdalin may represent a promising natural compound for further investigation as a potential therapeutic agent in neuroblastoma.

## 1. Introduction

Neuroblastoma is a malignancy of the sympathetic nervous system derived from differentiation-arrested neural crest cells. It is the most common extracranial solid tumor in children and is responsible for approximately one in six pediatric cancer-related deaths [1]. Most cases of neuroblastoma are sporadic, but the presence of genetic alterations including c-Myc amplification, found in about 20% of patients, can influence the risk of developing the disease [2]. Neuroblastoma presents a wide clinical spectrum, ranging from low-risk cases that can be cured with or without mild treatment, to high-risk forms that are fatal in approximately 50% of patients [3]. Over the past few decades, clinical trials combining high-dose chemotherapy with autologous stem cell transplantation, immunotherapy, and differentiating agents have resulted in significant improvements in 5-year survival for patients with metastatic neuroblastoma [2]. However, these therapeutic approaches for neuroblastoma are associated with severe side effects, including organ damage, anemia, impaired fertility, and the development of secondary malignancy. As a result, in pediatric oncology, neuroblastoma management continues to be a significant challenge, and novel potential therapy approaches are needed to mitigate both toxicity and the long-term side effects [4].

Apoptosis is a process essential for normal growth and development, playing a key role in early embryonic development and immune system development. It also contributes to maintaining tissue homeostasis and eliminating damaged or potentially harmful cells [5]. High-risk cases exert significant intra-tumor heterogeneity and often present alterations of these processes that contribute to chemotherapeutic resistance [1,6]. Accumulating evidence indicates that p53, Bcl-2 family proteins, and caspases play critical roles in neuroblastoma development [7]. c-Myc is a transcription factor that regulates proliferation, cell cycle, and apoptosis, and its amplification in neuroblastoma promotes cellular proliferation and apoptosis; however, this apoptotic signal is suppressed by reducing p53 activity, downregulating pro-apoptotic proteins or overexpressing anti-apoptotic proteins [8]. In preclinical models, the ErbB tyrosine kinases have also been demonstrated to induce cell growth and suppress apoptosis in neuroblastoma through the PI3K–AKT and MAPK–ERK pathways [9,10]. Neuroblastoma can spontaneously regress via cellular differentiation or reactivation of apoptosis, demonstrating how important apoptosis can be as a therapeutic target [8]. In this context, investigating the therapeutic potential of phytochemical compounds that can trigger tumor cell death may contribute to the development of new and effective approaches in the treatment of neuroblastoma [11].

Phytochemicals are considered invaluable and useful agents for cancer chemoprevention, because of their anticipated multimodal actions, limited toxicity, and minimal side effects. These compounds constitute a considerable portion of currently used chemotherapy agents, and many preclinical studies have confirmed their anticancer potentials and pro-apoptotic properties [12]. Amygdalin (IUPAC name: [(6-O-β-D-glucopyranosyl-β-D-glucopyranosyl)oxy](phenyl)acetonitrile) is a cyanogenic molecule in the aromatic cyanogenic glycoside class. It is a naturally occurring biomolecule that can be found in the prunasian family seeds (seeds of apricots, almonds, cherries, plums, peaches, apples, etc.) [13,14]. Amygdalin is emerging as a potent anti-neoplastic agent because of its chemoprevention and ability to induce apoptosis. The potential benefits of amygdalin in cancer prevention have been confirmed in lung [15], breast [16,17], prostate [18], and liver cancer [19] using in vitro studies. It has been reported to promote apoptosis in cervical cancer cells through mitochondrial-dependent pathways [20]. Another study has been demonstrated that amygdalin induces apoptosis via inhibiting NOX4/ROS/p38MAPK signaling pathway in mesenchymal stem cells [21]. The aim of this study is to demonstrate the dose- and time-dependent anti-proliferative activity of the amygdalin molecule in SH-SY5Y neuroblastoma cells in vitro and to elucidate the mechanism of action through changes in the expression of genes associated with apoptosis and invasion.

## 2. Materials and Methods

### 2.1. Cell Culture

Human neuroblastoma cells (SH-SY5Y) were cultivated in DMEM basal medium (Gibco; Gibco Laboratories, Grand Island, NY, USA) with the addition of 10% fetal bovine serum (FBS; FBS, Ebsdorfergrund, Germany; Capricorn Scientific GmbH, Ebsdorfergrund, Germany), 20 units/mL penicillin, 20 μg/mL streptomycin, and 0.1 mM non-essential amino acid solution (Gibco Laboratories, Grand Island, NY, USA). SH-SY5Y human neuroblastoma cell line used in our study was kindly provided by Dr. Semih Tan (Ordu University, Ordu, Türkiye) upon request.

The process of cell proliferation and preparation for the experiment is as described in the previous study [22]. Cells were maintained at 37 °C in a humidified atmosphere containing 5% CO_2_ under standard culture conditions. Upon reaching the appropriate level of confluence, the cells were subcultured and prepared for subsequent experimental analyses. This study evaluated the effects of amygdalin on cell viability at concentrations ranging from 100 to 1000 µM. These concentrations were selected based on the literature [16,17,18,19,20]. Amygdalin has been dissolved in DMSO at a concentration below 0.1%. Due to the low concentration (at a concentration of 0.5%), the effect of the solvent did not need to be taken into account. Each experiment was carried out in triplicate (*n* = 3) across three independent biological replicates.

### 2.2. Determination of Cytotoxicity Using the CCK8 (WST-8/CCK8) Assay

The cytotoxic effects of amygdalin were analyzed by CCK8 assay (Cell Counting Kit 8; WST-8; Abcam, Lot 1028841-1; Cambridge, UK) according to kit protocol. The SH-SY5Y neuroblastoma cells (3  ×  10^3^ cells/well) were cultured in 96-well plates in media for 24 and 48 h. Cells were treated with increasing concentrations of 100 μM, 200 μM, 400 μM, 600 μM, 800 μM and 1000 μM of amygdalin for 24 h and 48 h. After incubation, CCK solution was added to 96-well plates and incubated at 37 °C for 4 h. Subsequently, absorbance values (OD) were read at 450 nm wavelength using an ELISA reader (Biotek-EPOCH2; BioTek Instruments, Winooski, VT, USA). The half maximum inhibitory concentration (IC50) of nanoparticles was determined by using GraphPad Prism (version 9.4.1). The percentage of cell viability was determined using the formula given by Aybek et al., 2025 [23].

### 2.3. Real-Time PCR Analysis

SH-SY5Y neuroblastoma cells were treated with amygdalin at the IC50 dose, as determined by CCK8 assay or media only (control). Total RNA was isolated via TRIzol (Invitrogen, Waltham, MA, USA), following the protocol provided by the manufacturer. NanoDrop was used to measure the amount and quality of RNA (BioSpec-nano, Shimadzu, Japan). The High Capacity cDNA Synthesis kit with Rnase Inh. (ABT, Cat No: C03-01-20, Ankara, Turkey) was utilized for cDNA synthesis. Relative RNA levels were detected using the Rotor Gene 6000 Real-time PCR Thermocycler with WizPureTM qPCR master mix (SYBR, W1711, Bioneer Corp., Daejeon, Republic of Korea). The primer sequences for the amplification of human caspase-3, caspase-7, caspase-8, caspase-9, caspase-10, Bax, Bcl-2, Bid, NF-κB (p65), MMP-2, MMP-9, TIMP-1 and TIMP3 are presented in Table 1. The following cycle conditions were employed: initial denaturation at 95 °C for 15 min, followed by 15 s at 95 °C and 1 min at 60 °C for a total of 40 cycles. The expression fold-change for each target was calculated by the 2^−∆∆CT^ method, with GAPDH serving as the normalization control.

### 2.4. NF-Kappa B (NF-κB, Nuclear Factor Kappa B) ELISA

The NF-κB level of control and amygdalin-treated cells was investigated by an enzyme linked immunosorbent assay (ELISA) kit according to kit protocol (BT Lab, Cat No: E0690Hu; Shanghai, China). Control and dose group SH-SY5Y neuroblastoma cells were homogenized in RIPA lysis buffer including protease inhibitors cocktail and centrifuged at 12,000× *g* for 15 min at 4 °C. The supernatant was harvested and utilized for measuring NF-κB levels. The concentration of NF-κB (ng/mL) was determined by comparing the sample readings to a pre-established standard curve and was expressed in ng/mL.

### 2.5. Caspase-3 ELISA

The effect of amygdalin on the level of the caspase-3 protein, a significant apoptosis marker, in SH-SY5Y neuroblastoma cells was determined using the “Human Caspase-3 ELISA Kit, Cat. No. E4804Hu, BT LAB, Shanghai, China”. According to the manufacturer’s protocol, changes in caspase-3 protein concentration were determined in control cells and cells treated with amygdalin molecules. The absorbance for each well was recorded at 450 nm using a Biotek Epoch 2 microplate reader (BioTek Instruments, Winooski, VT, USA). All experiments were performed in triplicate. This method confirmed the gene expression changes observed in RT-PCR and the apoptotic efficacy of amygdalin.

### 2.6. Statistical Analysis

Real-time PCR results were analyzed using the ΔΔCt quantification approach, with data processing supported by the GeneGlobe RT-PCR Analysis RT^2^ Profiler PCR Array Data Analysis platform (Qiagen, Hilden, Germany). Statistical analyses were carried out using GraphPad Prism software version 9.4.1. Data are expressed as mean ± standard deviation (SD) based on a minimum of three independent biological experiments. Mean comparisons were performed using either an unpaired *t*-test or one-way analysis of variance (ANOVA), with Dunnett’s test applied for post hoc analysis. Statistical significance was defined as a *p*-value less than 0.05 (ns, *p* > 0.05; * *p* ≤ 0.05). Each experiment was carried out in triplicate (*n* = 3) across three independent biological replicates.

## 3. Results

### 3.1. CCK8 Assay

**Figure 1:**
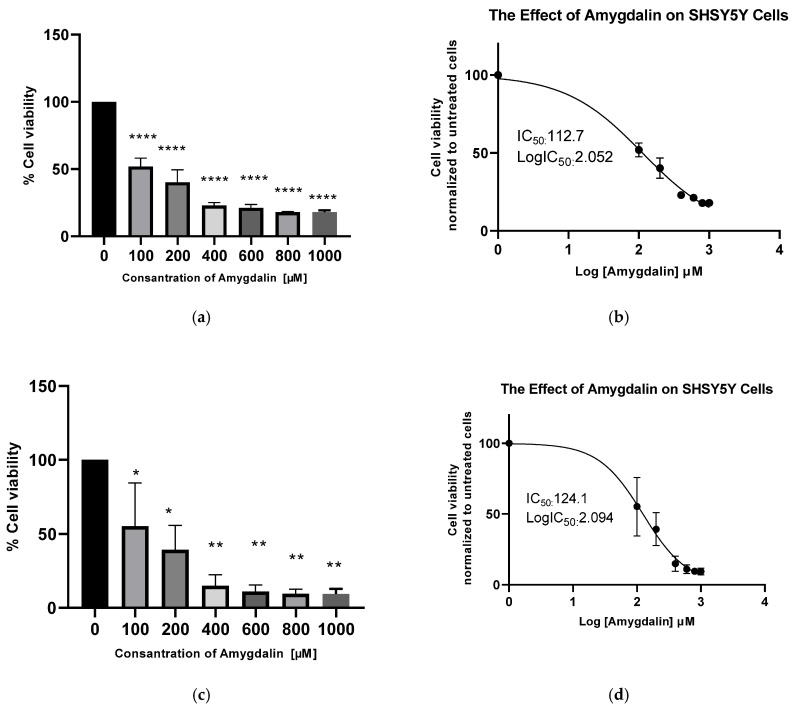
(**a**) The in vitro cytotoxic effect of amygdalin on SH-SY5Y cells after 24 h. (**b**) The IC50 value of amygdalin was determined to be 112.7 µM after 24 h. (**c**) The in vitro cytotoxic effect of amygdalin on SH-SY5Y cells after 48 h. (**d**) The IC50 value of amygdalin was determined to be 124.1 µM at 48 h. All experiments were conducted in triplicate (*n* = 3) across three independent biological replicates. Results are reported as the percentage of normalized cell viability (%) relative to control cells. Comparisons between means were performed using one-way ANOVA, and the Dunnett test was used for post hoc analysis (* *p* ≤ 0.05, ** *p* ≤ 0.01, and **** *p* ≤ 0.0001).

The cytotoxic effect of amygdalin on SHSY5Y neuroblastoma cells under in vitro conditions was determined using the CCK8 method based on colorimetric assay. Based on the results obtained, the IC50 value was determined using the GraphPad program for the 24 and 48 h results. As shown in Figure 1a,b, treatment with amygdalin at different concentrations ranging from 100 µM to 1 mM exhibited an anti-proliferative effect on SH-SY5Y neuroblastoma cells at 24 h. It was observed that amygdalin reduced cell proliferation in a dose-dependent manner, but this effect remained constant at high doses. The IC50 value of amygdalin in SH-SY5Y neuroblastoma cells at 24 h was determined to be 112.7 µM. Figure 1b shows a graph of the IC50 value.

Upon thorough examination of the data derived from the 48 h results of the CCK8 assay, a comparable scenario to that observed in the 24 h results is ascertained. Furthermore, at a 48 h time point, amygdalin was observed to reduce the proliferation of SH-SY5Y cells in a dose-dependent manner (Figure 1c). The IC50 value was determined to be 124.1 µM at 48 h (Figure 1d).

### 3.2. Real-Time PCR Gene Expression Assay

Real-time PCR experiments were conducted to investigate changes in the levels of mRNA expression of genes associated with apoptosis and invasion in SH-SY5Y cells following amygdalin administration. In comparison with the control group, the amygdalin-treated group exhibited statistically significant increases in apoptosis-related genes, including caspase-7, caspase-8, BID, and BAX, by 8.06-fold, 2.42-fold, 20.24-fold, and 3.29-fold, respectively (*p* < 0.005; Table 2). Furthermore, the expression of caspase-3 increased by 1.21-fold, and that of caspase-10 increased by 2.41-fold. However, these changes did not reach statistical significance. Furthermore, BCL-2 mRNA expression was found to be downregulated 10.17-fold in cells from the amygdalin-treated dose group. However, this change was not found to be statistically significant (*p* > 0.05). The expression level of NF-κB mRNA, an important marker of survival, decreased 3.03-fold in the amygdalin-treated group, and this change was statistically significant (*p* < 0.05). The results of this study suggest that amygdalin may exert pro-apoptotic and anti-proliferative effects in neuroblastoma cells, as evidenced by the modulation of apoptosis-related gene expression, including the upregulation of pro-apoptotic markers and downregulation of the anti-apoptotic gene BCL-2. These effects may be associated with NF-κB signaling modulation; however, a direct mechanistic link between NF-κB inhibition and apoptosis induction could not be established in the present study, and the findings should therefore be interpreted as preliminary.

**Figure 2:**
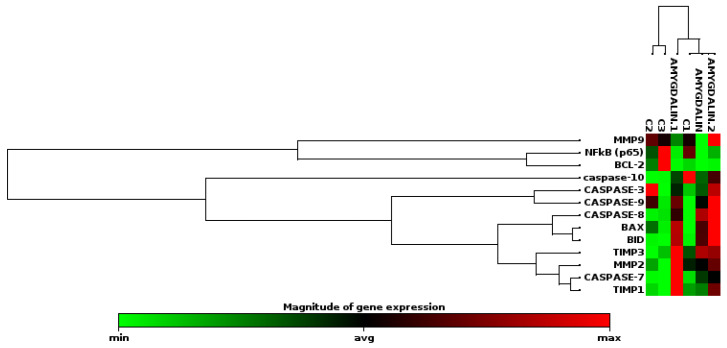
Clustergram heat map analysis in gene expression analysis of control and amygdalin-treated dose group samples (C: Control).

Furthermore, an examination of the expression changes of MMP-2 and MMP-9, matrix metalloproteinases associated with invasion, in SH-SY5Y cells treated with amygdalin, revealed no statistically significant change. However, the 1.82-fold decrease in MMP9 expression in the dose group cells is a significant indicator of reduced invasion. Furthermore, the mRNA expression levels of the MMP inhibitors TIMP1 (tissue inhibitor of metalloproteinases-1) and TIMP3 (tissue inhibitor of metalloproteinases-3) showed a 3.77-fold and 2.54-fold increase, respectively, in the amygdalin-treated dose group. The upregulation of TIMP3 is statistically significant (*p* > 0.05; Table 2). These results suggest that amygdalin exposure may exhibit anti-invasive activity by decreasing MMP-9 expression and increasing TIMP1 and TIMP3 expression in SH-SY5Y cells. The clustergram analysis map for ct values is presented in Figure 2.

### 3.3. ELISA for NF-κB and Caspase-3

**Figure 3:**
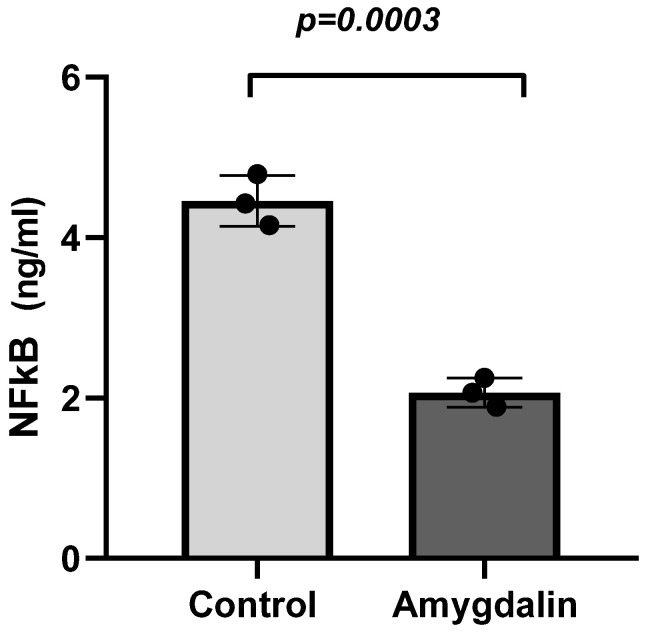
Comparative analysis between the amygdalin-treated group and the control group showed a significant decrease in NF-κB protein levels in neuroblastoma cells (*p* = 0.0003). The data are presented as the mean  ±  S.D. from at least three independent biological replicates. Comparisons between means were performed using unpaired *t*-test.

The protein concentrations of the control group SH-SY5Y cells and the amygdalin IC50 value-treated dose group cells were compared using the NF-κB ELISA test. Concentration calculations revealed that this value was 4.4 ng/mL in the analyzed control group, while it was 2.07 ng/mL in the amygdalin-treated dose group (Figure 3). And this decrease was found to be statistically significant (*p* < 0.05). These results show that the concentration of NF-κB, one of the cell survival markers, decreased in neuroblastoma cells as a result of amygdalin treatment. The decrease in mRNA levels observed in the real-time PCR experiments was also confirmed by ELISA.

**Figure 4:**
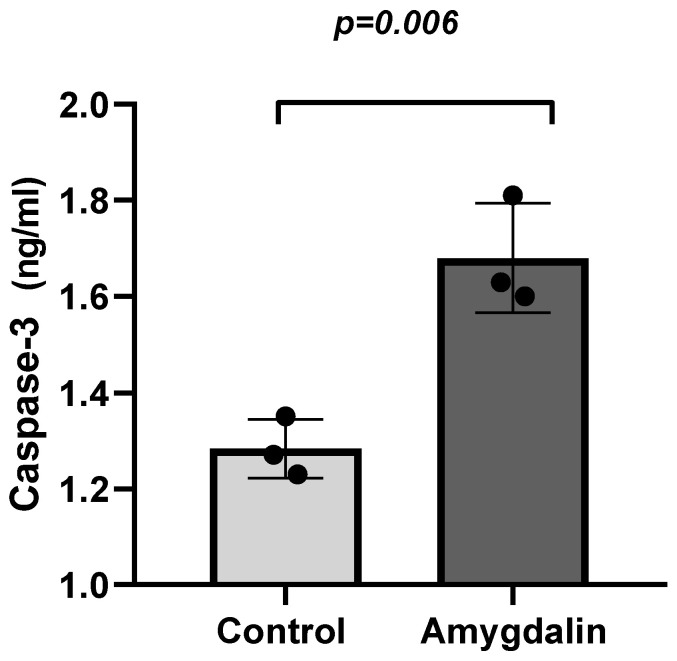
Comparative analysis between the amygdalin-treated group and the control group showed a significant increase in caspase-3 protein levels in neuroblastoma cells (*p* = 0.006). The data are presented as the mean  ±  S.D. from at least three independent biological replicates. Comparisons between means were performed using unpaired *t*-test.

To investigate the apoptotic efficacy of amygdalin, a study was conducted using the Caspase-3 ELISA method. According to the obtained results, the caspase-3 concentration in the control group of neuroblastoma cells was found to be 1.283 ng/mL, while the same concentration in the amygdalin-treated group was determined to be 1.680 ng/mL (Figure 4). Although there was an increase in the caspase-3 protein concentration in the nanoparticle-treated group, this change was not found to be statistically significant (*p* > 0.05).

## 4. Discussion

Neuroblastoma represents the most frequently diagnosed extracranial solid tumor of fetal origin in the pediatric population and constitutes approximately 7–10% of all childhood cancers [24]. Based on clinical and biological features, patients are stratified into very low-, low-, intermediate-, and high-risk categories. Nearly half of neuroblastoma cases are classified as high risk, and despite advances in current therapeutic strategies, the overall survival rate in this group remains below 50% [24,25]. Therefore, there is a critical need for the development of novel biological or genetically targeted treatment approaches that specifically address the molecular mechanisms driving tumor initiation and progression, particularly in high-risk neuroblastoma [25,26].

It is an established fact that for centuries, plants and their bioactive compounds have contributed to the management of various health conditions. Indeed, they have provided the foundation for many modern drugs derived from single active compounds. The increasing global demand for herbal medicines can be primarily attributed to their specific therapeutic benefits [27,28,29]. Furthermore, studies have reported that natural compounds may prevent neuroblastoma when used in isolation or in combination with other chemotherapy methods [29]. Amygdalin represents the main pharmacologically active compound in almonds and is also widely present in the seeds of plants belonging to the Rosaceae family. It exhibits several biological activities, including anti-inflammatory, antibacterial, anti-cancer, antioxidant, and immunomodulatory effects [30,31,32,33].

Our study demonstrated that amygdalin exhibits an anti-proliferative effect in SH-SY5Y neuroblastoma cells that is dependent on both dose and time under in vitro conditions. The IC_50_ dose was determined using the CCK8 method and found to be 112.7 µM after 24 h. To determine the molecular biological mechanisms underlying this effect, we investigated the impact on mRNA levels of genes associated with apoptosis and invasion. The results suggest that amygdalin may stimulate apoptosis in SH-SY5Y cells by upregulating the caspase-7, caspase-8, BID and Bax genes, while inducing downregulation of Bcl-2. Additionally, this study revealed that NF-κB expression, a key survival marker, decreased in neuroblastoma cells upon amygdalin exposure at both the protein and mRNA levels. This suggests modulation may have occurred via NF-κB -mediated signaling pathways. Furthermore, an increase in the concentration of the caspase-3 protein in neuroblastoma cells treated with amygdalin was also determined by ELISA. It has been confirmed that an increase in protein levels occurs in parallel with changes in gene expression. This finding suggests that amygdalin may induce apoptosis in SH-SY5Y neuroblastoma cells via caspase-3-mediated mechanisms. NF-κB is a key transcription factor involved in the regulation of cell survival, inflammation, and apoptosis. Under physiological conditions, NF-κB promotes cell survival by inducing the expression of anti-apoptotic genes such as BCL-2 and inhibitors of caspases. Conversely, inhibition of NF-κB signaling has been associated with the activation of apoptotic pathways and increased cellular susceptibility to programmed cell death. Therefore, the observed downregulation of NF-κB in this study may contribute to the modulation of apoptosis-related signaling pathways. However, this association should be interpreted cautiously, as a direct causal relationship between NF-κB inhibition and apoptosis induction was not experimentally confirmed in the present study.

A study has demonstrated that amygdalin exhibits an oxidative stress-mediated cytotoxic effect in T98G glioblastoma cells under in vitro conditions [34]. A study conducted on Hs578T Triple-negative breast cancer cells reported that amygdalin induces apoptosis by increasing the levels of pro-apoptotic proteins BAX and CASPASE-3 and decreasing the level of BCL-2, thereby exerting an anti-proliferative and anti-adhesive effect on the cells [35]. Notably, amygdalin has been shown to markedly induce apoptosis in both MCF-7 and MDA-MB-231 breast cancer cell lines by inhibiting cell proliferation and enhancing the effectiveness of radiotherapy via cell cycle arrest at the G1 and sub-G1 phases [36]. Amygdalin has been proposed as a potential adjunctive agent in the treatment of lung cancer. It has been shown to markedly promote apoptosis in A549 and PC9 lung carcinoma cells in a dose-dependent fashion through activation of the mitochondrial, caspase-mediated apoptotic signaling pathway [15]. The apoptotic response was accompanied by elevated cytochrome c release and increased activities of caspase-9 and caspase-3 in both A549 and PC9 cell lines. Additionally, suppression of cell proliferation in H1299/M and PA/M lung cancer cell lines under in vitro conditions was observed only at relatively high concentrations of amygdalin. The same study also reported NF-κB-mediated effects in the amygdala in both in vitro and in vivo models [15]. As demonstrated in earlier research, amygdalin has been found to demonstrate antiproliferative properties in cervical cancer cells, with the process of apoptosis being induced as a result. In HeLa cells, amygdalin treatment was associated with an increased Bax/Bcl-2 ratio and elevated caspase-3 activity, which together contributed to the enhancement of its pro-apoptotic effects in cervical cancer models [20].

Furthermore, the anti-invasive capacity of amygdalin was examined in this study through its effects on the mRNA levels of the MMP2, MMP9, TIMP1, and TIMP3 genes. Amygdalin has been demonstrated to reduce invasion in SH-SY5Y cells by decreasing MMP9 expression and increasing the expression of the MMP inhibitors TIMP1 and TIMP3. In a previous study, treatment with lower concentrations of amygdalin significantly suppressed the migratory and invasive capacities of H1299/M and PA/M lung cancer cells. Collectively, these findings suggest that amygdalin may serve as a promising agent with anti-invasive and anti-metastatic potential in lung cancer [37]. Moreover, amygdalin has been observed to reduce the migratory capacity of MDA-MB-231 cells to a greater extent than that observed in MCF-7 cells [38]. The suppressive effects of amygdalin on cellular growth, as well as on the expression of the differentiation-related markers E-cadherin and N-cadherin, were observed in renal cell carcinoma (RCC) cells following in vitro exposure of the A498, Caki-1, and KTC-26 cell lines [39].

This study has demonstrated for the first time the cytotoxic and apoptotic effects of amygdalin on neuroblastoma cells. The aim of this study was to provide initial in vitro evidence of the anti-proliferative effects of amygdalin in SH-SY5Y neuroblastoma cells, and to elucidate its potential mechanisms of action by examining changes in the expression of genes associated with apoptosis and invasion. The findings are intended to serve as a preliminary basis for more comprehensive future studies in this context. However, the present study has several limitations. These include the use of a single neuroblastoma cell line and the absence of experiments involving non-cancerous (healthy) control cell lines. Despite the extensive utilization and in-depth characterization of the SH-SY5Y model in the investigation of neuronal and cancer-related molecular mechanisms, it is important to note its potential limitations in fully capturing the biological heterogeneity associated with diverse genetic backgrounds, tissue origins, and epigenetic profiles. Consequently, the findings of this study should be interpreted within the context of this specific in vitro model. Future studies involving multiple neuroblastoma cell lines and complementary in vivo models are warranted to validate and extend the generalizability of these results.

Furthermore, several functional assays, such as Matrigel invasion and wound healing migration assays, were not performed in the present study. In addition, protein-level validation of gene expression changes by Western blot analysis could not be conducted. The involvement of different signaling pathways in the mechanism of action of amygdalin, including NF-κB-related downstream functional validation, was also not comprehensively investigated. Therefore, although NF-κB downregulation was observed at both mRNA and protein levels, a causal relationship between NF-κB inhibition and apoptosis induction could not be established.

Another important limitation is that apoptosis-related molecular analyses were performed only at the IC_50_ concentration; thus, comparative evaluation across different doses was not possible at the molecular level, which restricts the interpretation of dose-dependent effects. Although caspase-3 activity was assessed to provide functional confirmation of apoptosis, additional apoptosis validation assays such as Annexin V/PI staining or TUNEL analysis were not included due to budgetary and technical constraints. Moreover, advanced experimental approaches such as genomics-based analyses, organoid models, three-dimensional (3D) cell culture systems, in vivo validation studies, and pharmacokinetic evaluations were not performed in this study. These methodologies would provide a more comprehensive and translational understanding of the biological effects of amygdalin. Despite these limitations, the present study provides important preliminary in vitro evidence suggesting a potential role of amygdalin in modulating apoptosis-related pathways, including NF-κB signaling, in SH-SY5Y neuroblastoma cells. These findings may serve as a foundation for future studies employing more extensive functional, mechanistic, and in vivo validation approaches.

Amygdalin, which has historically been associated with laetrile, has long been the subject of scientific and clinical controversy, primarily due to its potential to release cyanide during metabolic degradation. This has given rise to significant safety concerns, particularly in systemic applications [40]. Whilst the present study demonstrates that amygdalin exerts anti-proliferative and pro-apoptotic effects in SH-SY5Y neuroblastoma cells under in vitro conditions, it is important to exercise caution when interpreting these findings. In vitro experimental systems are not capable of replicating the complexity of in vivo metabolism, pharmacokinetics, and toxicity profiles. Consequently, the concentrations utilized in this study may not directly correspond to physiologically achievable or safe levels in clinical settings. It is important to note that the current data do not provide sufficient evidence to support clinical utility. Further studies, incorporating in vivo models and comprehensive toxicological and pharmacokinetic evaluations, are necessary to ascertain the safety, efficacy, and translational potential of amygdalin.

## 5. Conclusions

The present study demonstrated that amygdalin exerts anti-proliferative and pro-apoptotic effects in SH-SY5Y neuroblastoma cells under in vitro conditions. Consistent with previous reports, amygdalin appears to modulate cellular processes associated with proliferation and apoptosis. In particular, the observed changes in apoptosis-related molecular markers, together with increased caspase-3 activity, suggest the activation of apoptotic signaling pathways at the cellular level. However, these findings should be interpreted with caution. Although NF-κB downregulation was observed at both mRNA and protein levels, a direct causal relationship between NF-κB inhibition and apoptosis induction could not be established within the scope of this study. Similarly, while caspase-3 activity supports apoptotic involvement, the current data are insufficient to definitively conclude a fully caspase-dependent mechanism. Furthermore, in vitro experimental conditions are unable to fully reflect the complexity of in vivo biological systems, including pharmacokinetics, metabolism, and systemic toxicity. The clinical applicability of amygdalin remains highly controversial due to the well-documented concerns regarding its metabolism and potential for cyanide release [41]. Despite neuroblastoma being a high-risk pediatric malignancy with limited treatment options in advanced stages, the present findings do not support direct clinical application of amygdalin. Instead, they should be considered preliminary in vitro evidence contributing to the understanding of its biological activity. The clinical use of amygdalin remains controversial due to concerns regarding its metabolism and potential cyanide release. Future studies should incorporate multiple cell lines, dose-dependent experimental designs, comprehensive functional assays, advanced molecular techniques, and in vivo models to elucidate the underlying mechanisms more clearly. Additionally, alternative strategies such as optimized delivery systems or structural modifications may be necessary to overcome its known limitations.

In conclusion, this study provides preliminary in vitro evidence of the biological effects of amygdalin in neuroblastoma cells and serves as a foundation for further mechanistic and translational research in this field.

## Figures and Tables

**Table 1:** Reverse and forward sequences of the primers.

Gene Name	Forward	Reverse
*GAPDH*	GTCTCCTCTGACTTCAACAGCG	ACCACCCTGTTGCTGTAGCCAA
*Caspase-3*	GGAAGCGAATCAATGGACTCTGG	GCATCGACATCTGTACCAGACC
*Caspase-7*	CGGAACAGACAAAGATGCCGAG	AGGCGGCATTTGTATGGTCCTC
*Caspase-8*	AGAAGAGGGTCATCCTGGGAGA	TCAGGACTTCCTTCAAGGCTGC
*Caspase-9*	GTTTGAGGACCTTCGACCAGCT	CAACGTACCAGGAGCCACTCTT
*Caspase-10*	CCAGGCTATGTATCCTTTCGGC	TCGTTGACAGCAGTGAGGATGG
*p65*(NF-κB)	TGAACCGAAACTCTGGCAGCTG	CATCAGCTTGCGAAAAGGAGCC
*BAX*	TCAGGATGCGTCCACCAAGAAG	TGTGTCCACGGCGGCAATCATC
*BCL-2*	ATCGCCCTGTGGATGACTGAGT	GCCAGGAGAAATCAAACAGAGGC
*BID*	TGGGACACTGTGAACCAGGAGT	GAGGAAGCCAAACACCAGTAGG
*MMP-2*	AGCGAGTGGATGCCGCCTTTAA	CATTCCAGGCATCTGCGATGAG
*MMP-9*	GCCACTACTGTGCCTTTGAGTC	CCCTCAGAGAATCGCCAGTACT
*TIMP-1*	GGAGAGTGTCTGCGGATACTTC	GCAGGTAGTGATGTGCAAGAGTC
*TIMP-3*	TACCGAGGCTTCACCAAGATGC	CATCTTGCCATCATAGACGCGAC

**Table 2:** The present study examined the alterations in apoptosis and invasion-related gene expression in SH-SY5Y cells exposed to amygdalin, utilizing real-time PCR as an analytical tool in comparison with the control group. GAPDH was utilized as a housekeeping gene for the purpose of normalization. The data pertaining to the amygdalin dose group was then compared with that of the control group, with the fold regulation and *p*-value metrics being utilized to facilitate this analysis (*: *p* < 0.05).

Gene Symbol	Amygdalin Dose (IC_50_) Group	
	Fold-Regulation	*p* Value
*Caspase-3*	1.21	0.651736
*Caspase-7*	8.06	0.033329 *
*Caspase-8*	3.89	0.002728 *
*Caspase-9*	2.42	0.110479
*Caspase-10*	2.41	0.789069
*BAX*	3.29	0.006087 *
*BCl-2*	−10.17	0.199737
*BID*	10.24	0.001288 *
*NF-κB*	−3.03	0.034708 *
*MMP-2*	1.62	0.054336
*MMP-9*	−1.82	0.595062
*TIMP1*	3.77	0.065930
*TIMP3*	2.54	0.003489 *

## Data Availability

The data sets and analyses from the study can be obtained from the corresponding and co-authors upon reasonable request.

## Abbreviations

The following abbreviations are used in this manuscript:

MMP	matrix metalloproteinase
NF-κB	Nuclear Factor kappa B
cDNA	Complementary DNA
TIMP	tissue inhibitor of metalloproteinases
